# *Plasmodium vivax* malaria at households: spatial clustering and risk factors in a low endemicity urban area of the northwestern Peruvian coast

**DOI:** 10.1186/s12936-015-0670-y

**Published:** 2015-04-24

**Authors:** Angel Rosas-Aguirre, Oscar J Ponce, Gabriel Carrasco-Escobar, Niko Speybroeck, Juan Contreras-Mancilla, Dionicia Gamboa, Edwar Pozo, Sócrates Herrera, Alejandro Llanos-Cuentas

**Affiliations:** Instituto de Medicina Tropical Alexander von Humboldt, Universidad Peruana Cayetano Heredia, Lima, Peru; Research Institute of Health and Society (IRSS), Université catholique de Louvain, Brussels, 1200 Belgium; Departamento de Ciencias Celulares y Moleculares, Facultad de Ciencias y Filosofia, Universidad Peruana Cayetano Heredia, Lima, Peru; Sub-región de Salud Luciano Castillo Colonna, Sullana, Peru; Caucaseco Scientific Research Center, Cali, Colombia

**Keywords:** Malaria, Hotspots, Clustering, Risk factors, Low transmission

## Abstract

**Background:**

Peru has presented a decreasing malaria trend during the last decade, particularly in areas on northwestern coast; however, a limited number of cases continues to be reported yearly mainly in malaria hotspots.

**Methods:**

A two-phase study was conducted to identify spatial and temporal clusters of incident *Plasmodium vivax* malaria, as well as to determine risk factors associated with households (HH) presenting *P. vivax* malaria episodes in an urban area of the northwestern Peruvian Coast from June 2008 to May 2010. In the first stage, a full census of the study population was conducted, including geo-referencing of reported *P. vivax* episodes. In the second stage, a population-based case–control study allowed the identification of risk factors associated with HHs reporting episodes. A total of 117 case HHs with reported *P. vivax* and 117 control HHs without malaria episodes were assessed. A semi-structured questionnaire was used to interview the head of households and to collect data on HH location and structure, availability of public services, preventive malaria measures, family member with outdoor occupation (farmer, moto-taxi driver), and other HH characteristics. Univariate and multivariate logistic regression analyses were performed to determine case-HH risk factors. SaTScan was used to detect spatial and temporal *P. vivax* malaria clusters.

**Results:**

The most likely spatial cluster of malaria incidence included 1,040 people (22.4% of total population) in 245 HHs (24.6% of total HHs) accounting for 283 malaria episodes (40.1% of total episodes) during the study period (RR = 2.3, p < 0.001). A temporal cluster was also identified from April 12, 2009 to July 4, 2009 accounting for 355 malaria episodes (50.4% of total episodes) (RR = 7.2, p = 0.001). Factors significantly associated with case HHs compared with control HHs were: proximity to water drain < 200 metres (OR = 2.3, 95% CI: 1.3, 4.0); HH size >5 individuals (OR = 1.8, 95% CI: 1.0, 3.2); lack of potable water (OR = 1.8, 95% CI: 1.1, 3.2); and having domestic and peridomestic animals (OR = 3.6, 95% CI: 1.3, 9.5).

**Conclusion:**

*Plasmodium vivax* malaria incidence is highly heterogeneous in space and time in the urban study area with important geographical and housing risk factors associated with symptomatic episodes.

## Background

Despite recent reduction, malaria remains the most important human arthropod-borne disease worldwide [1]. In Peru, the northern coast, which includes Tumbes and Piura departments, has historically been the second most important region for malaria transmission after the Amazon rainforest [2]; however, the significant decline in malaria incidence over the past decade has modified the epidemiological profile of the disease in the region [3]. Currently, all endemic areas in the northern coast are of low or very low malaria transmission.

**Figure 1:**
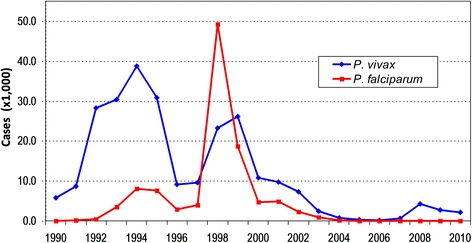
Annual malaria incidence in Piura department: 1990–2010.

Specifically, in Piura, after the malaria epidemic associated with the El Niño Southern Oscillation (ENSO) climatologic phenomenon in 1998 [4], when around 50,000 *Plasmodium falciparum* and 23,000 *Plasmodium vivax* malaria episodes were reported, malaria dropped drastically in the following years and since 2004 has reported variable incidence below 5,000 annual episodes (Figure 1). Between 2004 and 2010, *P. vivax* incidence fluctuated between 315 and 4,185 episodes annually and *P. falciparum* malaria has become scarce with no autochthonous cases reported in 2007, in 2009 nor in 2010 [5]. Many localities in Piura have not reported malaria during the past five years, and those that still report cases are primarily located in a few districts of Sullana province.

As transmission declines, it often becomes increasingly focal [6]. This demands changes in the malaria control interventions to more efficiently target the remaining parasite reservoirs. In areas with low malaria incidence, like Sullana, passive case detection (PCD) at health facilities could lead to the identification of the households (HHs) or the clusters of HHs with significantly more infections than others that are believed to be the source for new infections. Geo-referencing of the HHs where symptomatic malaria episodes originate would contribute significantly to defining malaria transmission “hotspots” [6-8]. Geographical and temporal analysis of these episodes using spatial-analysis tools would allow finer geographical localization of malaria clusters i.e. at the HH scale, and may greatly contribute to improving prevention and control efforts by optimizing the delivery of limited resources to higher-risk populations [9-11].

Based on successful malaria control experiences in Mexico [12], other *P. vivax* mono-endemic mesoamerican countries have included as operative strategy for targeted efforts the identification of HHs of high malaria risk within localities of low transmission [13]. HHs with any confirmed *P. vivax* infection in the previous three years are prioritized for active case detection (ACD), delivery of long-lasting insecticide treated nets (LLITNs), and indoor residual insecticide spraying (IRS) [13]. A similar operative approach has been used in some areas of the northern Peruvian coast during the implementation of ACD interventions; however, the annual decline in reported episodes is making less clear the stratification of malaria risk at the household level.

Besides spatial analysis, the identification of factors influencing malaria risk in HHs can also guide targeted interventions in low transmission settings [14]. Important risk factors such as housing type, house proximity to mosquito breeding sites, and others associated with the main economic activities and malaria preventive measures at the household level have been identified [6,15-17]. The present study aimed to identify spatial and temporal clusters of incident *P. vivax* malaria in three urban neighbourhoods of Bellavista district in Sullana-Piura between June 2008 and May 2010 as well as to determine through a simple case–control design the risk factors associated with HHs presenting *P. vivax* malaria episodes during the same period.

## Methods

### Study area

**Figure 2:**
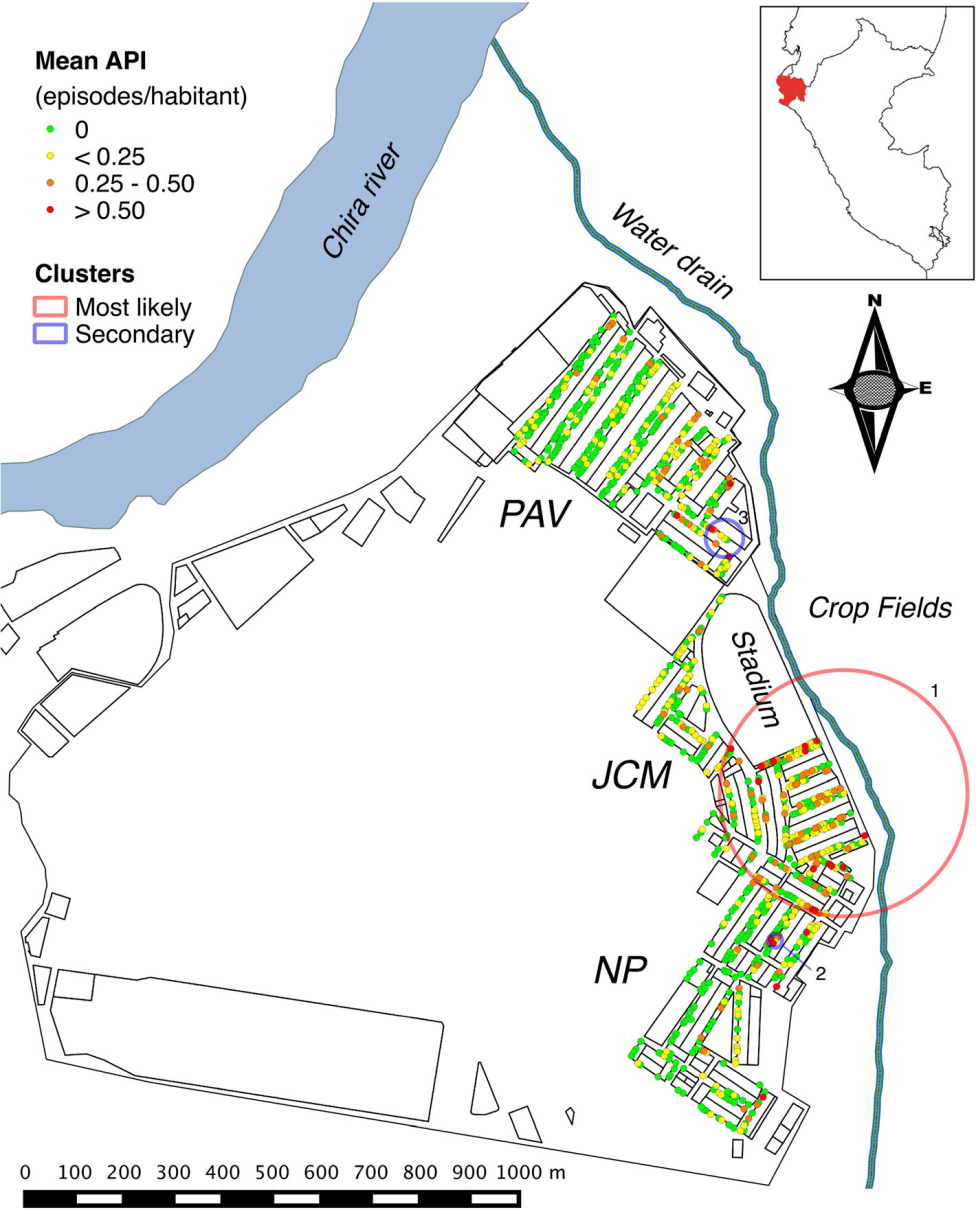
Significant *P. vivax* spatial clusters in Bellavista district.

Bellavista is a small urban district in Sullana province in Piura department on the northwestern Peruvian coast (Figure 2). It is located 30 km from Piura and is the fifth largest city in Peru. Bellavista is considered one of the most populated districts in the country with ~37,000 inhabitants living in an area of 3.09 km^2^. Three neighbourhoods that had reported the majority of the district’s malaria episodes in the past two years were selected. They are located in the eastern part of the district, from north to south: Pavletich (PAV) with nine blocks of houses; Jose Carlos Mariátegui (JCM) with 12 blocks and a big area occupied by the municipal stadium; and Nuevo Porvenir (NP) with 13 blocks.

Of note, the southern walls of the municipal stadium in JCM are bordered by dense emergent vegetation in front of which exists a block (with 55 individuals living in 16 HHs), that is the result of progressive illegal land invasion which has occurred over the past five years. At the time of the study, most of the houses in this block were constructed with perishable materials and lacked basic services. Moreover, the surrounding area was one of those in the district most affected by delinquencies.

Situated at the eastern edge of the study area, a large artificially created water drain flows south to north for about 2 km and empties into the Chira River. The drain width ranges between 3 and 100 metres and the depth is between 2 and 30 metres with the maximum dimensions reached at the mouth to Chira River. Patches of wild vegetation are observed on both sides of the drain and on the east side, a few metres beyond the drain, there is a large area with primarily seasonal fruit crops (lemon, mango, papaya, etc.).

In Bellavista, warm and dry winters range from May to October, while hot and humid summers with moderate rains occur from December to April. Malaria transmission is seasonal; the peak of cases occurs between February and May, the months in which the density of the main vector, *Anopheles albimanus*, increases [3,18,19]. However, this seasonal pattern has been periodically altered by the ENSO phenomenon [20] with torrential rains and strong winds causing flooding and landslides, which are associated with malaria outbreaks. Annual rainfall between 2008 and 2010 ranged between 82.8 mm and 193.5 mm, the average relative humidity between 71% and 76%, and the average temperature between 24.2°C and 24.8°C [21]. The main occupations in the area are informal trade, agriculture and small animal farming.

### Study design

A two-phase study was conducted. The first phase consisted of a full census of the study population and the identification and geo-referencing of the HHs of all reported *P. vivax* episodes in the study area between June 2008 and May 2010. The second phase was a population-based case control study to identify risk factors associated with HHs reporting any *P. vivax* episode during the same period.

### Census and geo-referencing of malaria episodes

The census, conducted in June 2010, included collection of sociodemographic and malaria history data. Each HH was identified with a unique code and geo-referenced using a handheld global positioning system (GPS) device (Garmin’s GPSMAP 60CSx, Garmin International Inc., USA). Presence/absence of malaria episodes during the study period was confirmed in all censored individuals, including those not reporting past malaria episodes, through the systematic revision of PCD records at the two nearby health facilities: Bellavista health post and Sullana Hospital. Moreover, a cross-checking of the census database (using names, age, gender and address) against the malaria surveillance database of the Sub-Regional Health Direction of Sullana (SRHDS) was performed to identify additional episodes registered in other health facilities. According to national guidelines, a malaria episode occurs in an individual with fever (body temperature >37.5°C) and/or history of fever in the previous two weeks in a malaria risk area and a thick blood smear positive for *Plasmodium* [22]. Immediately after *P. vivax* malaria is confirmed, individuals receive directly observed therapy with chloroquine (25 mg/kg over 3 days) and primaquine (3.5 mg/kg over 7 days). The easy access to malaria diagnostic testing in health facilities as well as the high awareness of malaria and appropriate treatment-seeking behaviour in the study population facilitate the detection of most of malaria episodes in the study area.

### Case–control study

The census data was used as a sampling frame for case and control HH selection. Cases were selected from all HHs with one or more *P. vivax* episode detected between June 2008 and May 2010 and controls from all HHs without an episode detected during the same period.

Sample sizes were calculated using the StatCalc module of EpiInfo (EpiInfo™ version 3.5.1, U.S. Centers for Disease Control and Prevention, Atlanta, GA, USA). The study was powered to detect an odds ratio of 2.2 with 95% significance and 80% power on the measure of having an assessed risk factor, assuming that 25% of HHs without malaria episodes had the risk factor, and a case to control ratio of 1:1. A total of 234 HHs were included: 117 case-HHs (with one or more malaria episodes) and 117 control-HHs (without malaria episodes) selected through stratified random sampling proportional to the total number of HHs in each neighbourhood.

Each selected HH was visited in June 2010 and a written informed consent (IC) was sought from the head of HH and members. If HH participants were absent at the time of the visit, the study team returned within two days to maximize subject participation. A semi-structured questionnaire was used to interview head of HHs and to collect HH location; housing structure; availability of essential services; preventive malaria measures; family member with outdoor occupation; and other HH characteristics. In addition, each available HH member was examined for fever and other malaria symptoms and a finger-prick blood sample was taken for immediate microscopy and further analyses. Malaria infected individuals were treated according to the national guidelines. Serological and molecular tests were performed at Institute of Tropical Medicine Alexander von Humboldt, Lima (ITM-AvH) using dried blood samples collected on filter paper (Whatman grade 3, Whatman, Springfield Mill, USA) which were stored at 4°C until use.

### Laboratory procedures

#### Microscopy

Thick and thin smears were stained for 10 min with a 10% Giemsa solution and parasite density was expressed as the number of parasites/μl after counting a total of 200 white blood cells (WBC) (or 500 WBCs if <10 parasites/field) and assuming an average of 6,000 WBC/μl according to the national guidelines [23]. Microscopy examination was performed immediately after sample collection at the reference laboratory in Sullana and blind quality control was independently performed at a later time on all positive slides and 10% of randomly selected negative slides by a senior technician at ITM-AvH. Any discordant results were reread by a second senior technician until agreement.

#### Species-specific polymerase chain reaction (ss-PCR)

Parasite DNA was extracted using the saponin Chelex 100 method [24]. Briefly, filter-paper blood spots containing ~20 μl of blood were cut into pieces of ~5 mm^2^ and incubated with 20 μl of 0.05% saponin at room temperature for four hours. Then, 10 μl of 20% Chelex 100 solution was added and the sample was incubated for 10 min at 95°C followed by a centrifugation at 11,000 g. The supernatant (DNA) was transferred into a new tube and stored at −20°C until PCR was performed. The DNA was amplified by a semi-nested multiplex PCR method targeting the 18S rDNA region as described by Rubio *et al.* [25]. PCR products were analysed in a 2% agarose gel with a standard 100 bp DNA ladder using ethidium bromide staining (0.5 μg/ml) and a data image analyzer with UV trans-illuminator.

#### Serology

Antibodies were eluted from filter paper spots and assayed in duplicate for specific IgG responses to *P. vivax* merozoite surface protein119 (*Pv*MSP1-19) using ELISA [26,27]. Samples were read at 405 nm and optical density (OD) values were corrected by subtracting the mean OD of the antigen negative control wells from the mean OD of the corresponding antigen containing wells. Analyses with duplicate OD values differing by more than 1.5-fold were rejected and rerun. To ensure sample result standardization across ELISA plates, percent positivity (PP) of each specimen was calculated using the OD of the positive control serum as 100%. Blind quality control was performed at ITM-AvH on 5% of randomly chosen samples. The criterion for positivity was determined by applying a mixture model to the PP data assuming two inherent normal distributions: a narrow distribution of seronegatives and a broader distribution of seropositives. The cut-off value was calculated as the mean plus 3 standard deviations of the narrow distribution [28].

### Data analysis

#### Temporal and spatial cluster analysis

Data were double entered, validated and cleaned in Excel (Microsoft Corp, USA). QGIS™ software v.2.0 [29] was used to map all census HHs with malaria episodes. The two-year mean annual parasite incidence (API) at the household level was calculated by dividing the mean annual number of reported *P. vivax* episodes in a HH during the study period by the total number of HH habitants. Using the API, HHs were classified according to four categories: 0 episodes/habitant, <0.25 episodes/habitant, 0.25-0.50 episodes/habitant, and >0.50 episodes/habitant. SaTScan^TM^ software v.9.3 [30] was used to detect spatial clusters of malaria incidence in the study area using the following settings: purely spatial analysis [31]; Poisson probability model; latitude/longitude coordinates; report of most likely clusters with no geographical overlap of secondary clusters; Gini optimizer cluster collection; and maximum spatial cluster size equal to 50% of total population. SaTScan applies multiple circular windows, which are plastic in both position and size, across the study area. Each circle represents a possible cluster of malaria incidence. Clusters were assessed based on 999 Monte Carlo simulations to determine the probability of the observed number of episodes being due to chance relative to the expected number of episodes. The null hypothesis of no clustering was rejected if any resulting p-value of assessed clusters was <0.05 and the window with the highest log-likelihood ratio (LLR) was identified as the most likely cluster. The relative risk (RR) reported for each identified cluster was the estimated risk within the cluster divided by the estimated risk outside the cluster [31].

In addition, a temporal analysis was carried out to identify temporal clusters of malaria incidence during the study period using the following settings: purely temporal analysis [31]; Poisson probability model; maximum temporal cluster size equal to 50% of the study period; and a time aggregation of one week. SaTScan establishes different windows (i.e. intervals) that move in only one dimension (time). The risk of malaria is then tested within and outside the time-interval window. The null hypothesis that the incidence was equally distributed during the study period was rejected if any resulting p-value of time interval clusters was < 0.05.

#### Risk factor analysis

Continuous variables were compared using the analysis of variance (ANOVA) or Mann–Whitney test (skewed data). For categorical variables, Chi-squared or Fisher’s exact tests were used to assess significant differences in proportions. All reported p-values are two-sided and were considered statistically significant if <0.05. Univariate and multivariate logistic regression analyses were performed to determine risk factors for case-HHs adjusting for all potential confounders such as: house location in malaria spatial clusters; distance to the water drain; HH size; predominant materials in walls and floor; availability of essential services (i.e. potable water, sewage system and electricity); ownership of animals; bed net ownership and use; and family member with outdoor occupation. Factors with p-values <0.2 for the likelihood ratio test in the univariate analysis were considered for inclusion in the multivariate adjusted model. Using the manual backward methods, the final model retained all variables that were significantly associated with case-HHs (p <0.05). The criterion for model selection was based on the Akaike information criterion (AIC) [32].

#### Ethical considerations

Permission was received from health and local authorities after explaining the purpose and procedures of the study. Signed IC was obtained prior to participation and blood sampling by all adults and the parents of all participating children <18 years of age. In addition to their parent’s consent, children more than seven years old provided signed informed assent prior to participation. Ethical clearance was obtained from the Ethics Review Board of the Universidad Peruana Cayetano Heredia, Lima, Peru (SIDISI code: 056736).

## Results

A total of 996 HHs and 4,650 individuals were identified during the census (~13% of the total population of Bellavista district), which were distributed over the three study neighborhoods as follows: PAV (433 HHs; 2,163 individuals), JCM (312; 1,358) and NP (251; 1,129). Sample sizes for the case–control study were as follows: PAV (100 HHs; 567 individuals), JCM (84; 390) and NP (50; 238) (Table 1). Analysis of PCD records and surveillance databases for the period June 2008 - May 2010 identified 705 registered *P. vivax* malaria episodes (42.4% of the total cases of Bellavista), which occurred in 616 individuals living in 401 HHs (40.3% of total censored HHs): 539 (87.5%) individuals with unique episodes, 66 with two episodes (10.7%), 10 (1.6%) with three episodes, and one with four episodes.

**Table 1:** **Selected households (HHs) for the case–control study**

		**Control HHs**		**Case HHs**		**Total**	
	**Neighbourhood**	**n**	**%**	**n**	**%**	**n**	**%**
**Total HHs**	PAV	285	47.9	148	36.9	433	43.5
	JCM	143	24.0	169	42.1	312	31.3
	NP	167	28.1	84	20.9	251	25.2
	Total	595	100.0	401	100.0	996	100.0
**Selected HHs for case-control study**	PAV	50	42.7	50	42.7	100	42.7
	JCM	42	35.9	42	35.9	84	35.9
	NP	25	21.4	25	21.4	50	21.4
	Total	117	100.0	117	100.0	234	100.0

The plot of the mean HH annual parasite incidence (API) showed that most HHs with the high *P. vivax* malaria incidence (API ≥ 0.25) were located in the central and eastern parts of the study area (Figure 2). Purely spatial analysis by SaTScan confirmed that malaria episodes were not randomly distributed. The most likely spatial cluster of malaria incidence was a 250 m radius cluster composed of 1,040 people (22.4% of total population) in 245 HHs (24.6% of total HHs) (LLR = 55.8, RR = 2.3, p < 0.001). Located south of the municipal stadium and including only HHs of the JCM neighbourhood, the cluster accounted for 283 reported malaria episodes (40.1% of total episodes during the study period) in 230 individuals (185 with one episode, 37 with two, and 8 with three). In addition, two small secondary clusters were identified: one located south of the most likely cluster composed of 38 people in 8 HH (LLR = 16.2, RR = 4.28, p < 0.001) accounting for 24 malaria episodes in 19 individuals (15 with one episode, three with two, and one with three) and the other cluster was located northeast of the stadium and was composed of 30 people also in 8 HHs (LLR = 10.1, RR = 3.81, p = 0.018) accounting for 17 episodes in 13 individuals (nine with one episode, and four with two). Moreover, during the study period, purely temporal analysis identified a 13-week temporal cluster of malaria incidence from mid-April (April 12th) to the first week of July (July 4th), 2009 accounting for 355 malaria episodes (50.4% of total episodes) (LLR = 299.2, RR = 7.2, p = 0.001).

Within the sampling frame of 401 HHs with symptomatic episodes and 595 HHs without episodes, 117 case-HHs and 117 control-HHs were selected, respectively. Case-HHs accounted for 196 registered *P. vivax* episodes in 171 individuals: 147 individuals with unique episodes, 23 with two episodes, and one with three episodes. Most of the case HHs (67; 57.3%) had only one malaria episode during the period, 32 (27.3%) case HHs had two episodes, and 18 (15.4%) had three or more episodes. Two or more episodes in a case-HH could be either in the same or in different individuals. There were no significant differences between case and control HHs with respect to head of household sociodemographic characteristics; i.e. mean age, gender, education level, main economic activities, and monthly salary (p > 0.05) (Table 2). Moreover, no differences between case and control-HHs were found with respect to the gender distribution and the mean age at the HH level (Table 3); in contrast, case-HHs had more family members than control-HHs (p = 0.03).

**Table 2:** **Sociodemographic characteristics of heads of HH**

			**Control HHs (N = 117)**		**Case HHs (N = 117)**		**p-value**
**Age in years**		mean (SD)	37.5	(12.5)	38.6	(11.7)	0.49
**Gender**		n (%)					
	Female		72	(61.5)	70	(59.8)	0.79
	Male		45	(38.5)	47	(40.2)	
**Education**		n (%)					
	None		3	(2.6)	5	(4.3)	0.49
	Incomplete primary		11	(9.4)	19	(16.2)	
	Complete primary		48	(41.0)	45	(38.5)	
	Secondary		49	(41.9)	44	(37.6)	
	Superior, university		6	(5.1)	4	(3.4)	
**Current economic activity**		n (%)					
	None		9	(7.8)	9	(7.7)	0.86
	Farmer		2	(1.7)	6	(5.1)	
	Trader		16	(13.8)	13	(11.1)	
	Moto driver		8	(6.9)	8	(6.8)	
	Hand labourer		20	(17.2)	22	(18.8)	
	Housewife		57	(49.1)	54	(46.2)	
	Other		4	(3.4)	5	(4.3)	
	Missing		1				
Monthly income amount*		n (%)					
	None		61	(55)	64	(56.6)	0.91
	<125 USD		18	(16.2)	16	(14.2)	
	≥125 USD		32	(28.8)	33	(29.2)	
	Missing		6		4		

*Income expressed in United States dollars (USD).

**Table 3:** **Characteristics of case and control HHs**

		**Control HHs (N = 117)**		**Case HHs (N = 117)**		**p-value**
**Number of individuals**	mean (SD)	4.8	(1.8)	5.4	(2.0)	0.03*
**Male percentage (%)**	mean (SD)	48.3	(19.1)	48.0	(16.4)	0.92
**Age, mean**	mean (SD)	23.8	(10.4)	23.2	(7.0)	0.59
**Number of tested individuals** ^∞^	mean (SD)	3.2	(1.6)	3.6	(1.7)	0.05
**% of tested individuals** ^∞^	mean (SD)	68.4	(23.3)	69.5	(22.0)	0.72
***P. vivax*** **Positive PCR**	n (%)	0	(0.0)	3	(2.6)	0.08
**% of individuals with** ***P. vivax*** **positive serology** ^**^**^	mean (SD)	8.1	(19)	22.2	(24.4)	<0.001

^∞^Tested using PCR and ELISA.

^^^Of the total tested individuals.

*p < 0.05.

In total, 795 individuals were tested by microscopy, PCR and serology: 421 in case-HHs (3.6 tested per HH) and 374 in control-HHs (3.2 tested per HH). Although there were no positive individuals by microscopy, three individuals were PCR positive to *P. vivax* (all in case-HHs). In addition, the percentage of individuals with positive *P. vivax* serology in case-HHs (22.2%) was almost three more times than that in control-HHs (8.1%).

In the univariate analysis, distance to water drain (p = 0.002); material in walls different than concrete or brick (p = 0.042); lack of potable water (p = 0.003); high bed net ownership (p = 0.033); and having domestic (i.e. cats, dogs) or peri-domestic (i.e. fowl, pigs) animals around the HH (p = 0.006) were significantly associated with case-HHs as compared with control-HHs (Table 4); and HH size (p = 0.07) reached the p-value to be included in the initial multivariate model. The multivariate model showed that four risk factors remained independently associated with case-HHs: distance to water drain ≤200 metres (OR = 2.3, 95% CI: 1.3, 4.0); HH size ≥ 5 individuals per HH (OR = 1.8, 95% CI: 1.0, 3.2); limited availability of potable water (OR = 1.8, 95% CI: 1.1, 3.2); and having domestic and peridomestic animals around the HH (OR = 3.6, 95% CI: 1.3, 9.5).

**Table 4:** **Risk factors for case-HHs**

		**Control HHs (N = 117)**		**Case HHs (N = 117)**		**Unadjusted**		**Adjusted** ^**+**^	
		**%**	**n**	**%**	**n**	**OR**	**95% CI**	**OR**	**95% CI**
**HH in malaria spatial cluster identified by Satscan**									
	No	74.4	87	70.9	83	1.0			
	Yes	25.6	30	29.1	34	1.2	[0.7;2.1]		
**House distance to water drain**									
	>200 metres	67.5	79	47.0	55	1.0		1.0	
	≤200 metres	32.5	38	53.0	62	2.3*	[1.4;4.0]	2.3*	[1.3;4.0]
**HH size**									
	≤5 individuals	71.8	84	60.7	71	1.0		1.0	
	>5 individuals	28.2	33	39.3	43	1.7**°**	[0.95;2.9]	1.8*	[1.0;3.2]
**Main material in walls**									
	Concrete, brick	34.2	40	22.2	26	1.0			
	Other material (wood, adobe, etc.)	65.8	77	77.8	91	1.8*	[1.0;3.3]		
**Main material in floor**									
	Cement or other fine finish	27.4	32	30.8	36	1.0			
	Other material (soil, stone, etc.)	72.7	85	69.2	81	0.8	[0.5;1.5]		
**Potable water availability**									
	Permanent (every day, all day)	62.9	43	43.0	49	1.0		1.0	
	Not permanent/ No availability	37.1	73	57.0	65	2.3*	[1.3;3.8]	1.8*	[1.1;3.2]
**Bathroom connected to sewage system**									
	Yes	53.1	60	50.0	58	1.0			
	No	46.9	53	50.0	58	1.1	[0.7;1.9]		
**Electricity available**									
	Yes	91.3	105	90.4	103	1.0			
	No	8.7	10	9.7	11	1.1	[0.5;2.8]		
**Has domestic** ^†^ **or peridomestic animals** ^**∞**^									
	No	94.7	108	82.5	94	1.0		1.0	
	Yes	6.3	6	17.5	20	3.8*	[1.5;9.9]	3.6*	[1.3;9.5]
**Bed net ownership**									
	None	39.3	46	26.5	31	1.0			
	>2 individuals/bed net	35.9	42	39.3	46	1.6	[0.9;3.0]		
	≤2 individuals/bed net	24.8	29	34.2	40	2.1*	[1.1;4.0]		
**Bed net use by head of HH**									
	Always	65.8	77	62.4	73	1.0			
	Never/Sometimes	34.2	40	37.6	44	1.2	[0.7;1.9]		
**Any HH member with outdoor occupation the past year** ^**^**^									
	No	88.0	103	84.6	99	1.0			
	Yes	12.0	14	15.4	18	1.3	[0.6;2.8]		

^+^Risk factors with p < = 0.2 were included in the multivariate logistic regression analysis.

*p < 0.05.

°p > 0.05 and <0.2.

^†^Dogs, cats.

^∞^fowl, pig.

^^^Farmer, moto-taxi driver.

## Discussion

*Plasmodium vivax* malaria episodes detected by PCD at health facilities between June 2008 and May 2010 were clustered in groups of HHs (hotspots) within the selected neighbourhoods of Bellavista district in the northern Peruvian coast. The main hotspot was located in the central part of the study area, bordered in the north by the southern side of the municipal stadium and in the east by the water drain. Proximity to the water drain and household characteristics such as family size, limited availability of potable water, and animal ownership were important risk factors for malaria HHs.

In areas of low malaria transmission with seasonal patterns affected by climatic conditions, malaria transmission is highly heterogeneous and consequently malaria acquisition risk for individuals is unevenly distributed even within a neighborhood [8] as in this study area. Even though Bellavista had already been recognized for malaria hotspots by the Ministry of Health (MoH) based on routine surveillance data (i.e. reported number of malaria episodes) [5], the combination of geographical systems (GIS) and spatial statistics allowed the identification of HHs clusters within the same neighbourhoods which are 2.3 and 4.8 times more likely to have reported *P. vivax* episodes compared with HHs outside the clusters. In addition, these tools detected a malaria outbreak reported in 2009 [3] whose magnitude extended the historical period of high transmission until July despite the implementation of control interventions (mainly indoor residual insecticide spraying). Actually, the number of cases reported during the identified temporal cluster (i.e. between April and July 2009) reached >50% of total reported *P. vivax* cases in the study period.

The location of the most likely spatial cluster of *P. vivax* malaria incidence may be explained by its proximity to a large drain where water frequently accumulates after the rains, thus providing favorable breeding habitats for *Anopheles* mosquitoes [18]. The irregularity in the bed and course of the drain as well as the waste disposal along it (common practice in the study area due to absence of a municipal waste collection service) facilitates water accumulation and proliferation of breeding sites mainly of *An. albimanus* in some sections of the drain near the cluster area. In this way, vegetation along the drain and that which borders the southern walls of the stadium appear to play an important role as refuge and resting places for adult mosquito populations [33].

Besides the proximity to vector breeding sites and vegetation, the biological features of *P. vivax* infections should also be considered among the possible explanations for the micro-geographical distribution of the disease, particularly the parasite ability to relapse weeks or months after a primary parasitaemia [34,35]. Indeed, the study showed that while the identified clusters accounted for 209 (28.8%) individuals of the total 539 individuals with unique episodes in the study population, the same clusters accounted for 53 (68.8%) individuals of the total 77 individuals with repeated episodes. Even though *P. vivax* relapse patterns may influence clustering of *P. vivax* episodes, the characterization and prediction of these patterns remain challenging due to the difficulty to distinguish between a hypnozoite-triggered relapse, a resurgence of erythrocytic parasites (i.e. recrudescence) due to a failure in the directly observed therapy, or reinfection of an individual with a new parasite strain following a primary infection [34,35]. This challenge is even greater in areas of low transmission where most infections are asymptomatic and sub-microscopic [5,18], and hence remain as undetected infections by the routine surveillance.

Although there is no unique explanation for the independent associations of the risk factors (distance to the water drain, family size >5 members, ownership of domestic and peri-domestic animals, and limited availability of potable water) which were significantly associated with *P. vivax* HHs, a common pathway between these HH risk factors and the occurrence of malaria infections may be a higher exposure to infectious mosquitoes following an increase of vector densities and/or of human-vector contact rates [8].

As mentioned above, water accumulation in the drain provides seasonal breeding sites between February and June that increases the size of mosquito population [8,18], resulting in a higher risk of being bitten by infectious *Anopheles* if the parasite is circulating in the area; people living closer to the drain would have higher exposure to the infectious vectors. On the other hand, an increased risk for malaria infections in bigger families has been described in areas where *P. falciparum* is predominant [36,37]. Although a case–control study in a residual transmission area of Mexico, which did not include the family size variable in the individual risk factor analysis for *P. vivax* infections, sleeping with two or more people in the same bed was independently associated with malaria risk [38]. Moreover, a recent ecological study which included >200 countries found a strong association between the reduction of the average household size and success in malaria control (after controlling for all commonly studied explanatory variables). This effect held across all climate zones irrespective of control measures, vector species, and *Plasmodium* species [39]. The effect of family size on malaria risk may be explained by the increase in mosquito attraction due to an increase in human-released chemo-attractants in reduced spaces [38]. Mosquitoes identify and find their hosts primarily through olfaction [40] and substances such as lactic acid and ammonia (presented in the human skin) and carbon dioxide (CO2) gas (exhaled through breathing) may be strong mosquito attractants [41].

The complex association between poverty and malaria is well known, which likely operates in both directions [42]: poor households are less able to afford prevention or treatment and the higher burden of malaria may push these same individuals deeper into poverty. Although reduced access to potable water reflects poor economic household conditions; the limited availability of this basic essential service itself may reduce the frequency of personal hygiene events (such as hand-washing and bathing) and clothing-washing practices [43], increasing the mosquito-attracting compounds on the skin and clothes and consequently the risk of being bitten by infectious mosquitoes [41]. Similarly, animal ownership may also increase malaria risk in HHs by increasing the attraction of zoophilic mosquitoes (under stimulus of CO2 and octenol) and consequently the human-vector contact rate [44]. Entomological observations have found that the host availability and other ecological conditions can influence the host choice of *An. albimanus* [19,45] whose feeding behavior ranges from highly anthropophilic to highly zoophilic [45]. A host preference study conducted in Belize indicated that *An. albimanus* seemed to prefer large animal mammals such cattle and pigs to most other host species (including human) [46], while other observations indicated that the vector can bite dogs, cats, goats, donkeys and even fowls [47].

The study has some limitations: first, the validity PCD data for the spatial identification of hotspots of high transmission intensity could be affected by an unequal spatial distribution of undetected asymptomatic infections following a differential acquisition of immunity inside and outside hotspots [6]. Although serological responses and parasite carriage indicators (including symptomatic and asymptomatic infections) are likely to produce most robust results in low endemic settings [48,49], they require more resource-intensive and expensive methods of collection which are not currently incorporated in the Peruvian routine surveillance system. Second, undetected asymptomatic infections during the study period, estimated to be 40% of the total infections in other endemic area of Peru [50], may have influenced the risk factor analysis, especially if those infections appeared more often in control-HHs than in case-HHs. However, serological results indicating that case-HHs had higher exposure to *P. vivax* parasites than control-HHs decreases the probability of this unequal allocation. Third, the study focused mainly on the identification of household risk factors associated with the occurrence of malaria episodes and did not consider individual risk factors that could also influence this HH condition. Fourth, even though a matched case–control study design was not used to control confounding, the stratified random sampling proportional to the total number of HHs in each neighbourhood provided balanced groups in regards sociodemographic characteristics and subsequent inclusion in identified spatial clusters. Finally, it must be considered that a *P. vivax* episode can be caused by either a relapse following a primary infection or a new infection (conditions that are difficult to distinguish from one another) [34,35] and each condition could have specific associated risk factors.

## Conclusion

Implementing targeted malaria control measures in areas of low incidence with patchy distribution and seasonal transmission, such as in the northwestern Peruvian coast, poses a major challenge to national malaria control programmes. In the study area, the availability of PCD surveillance and GPS data and the use of spatial analysis tools allowed the detection of groups of HHs (hotspots) where *P. vivax* malaria episodes are most likely to occur. A simple case–control design found that HHs with malaria episodes are more probable to have certain factors (HH risk factors) that may increase the family member’s risk of being bitten by an infectious *An. albimanus*. Integrated control strategies with participation from the local government and the community should be aimed to reduce identified risk factors by: increasing the accessibility to essential services (i.e. potable water, waste disposal services), avoiding and eliminating seasonal breeding sites and surrounding vegetation (i.e. community-based environmental management), decreasing behaviours and practices that increase exposure to the vector through effective communication campaigns, and incorporating the spatial analysis and risk factor monitoring within malaria surveillance activities. In summary, the combination of spatial, temporal, and risk factor analyses using routine data can improve malaria risk stratification and help decision-makers to better design and deliver targeted interventions. This is particularly important in low transmission settings where control efforts are intended to maintain low incidence levels, avoid outbreaks, and to achieve malaria-free zones as initial steps for malaria elimination in the country. Currently, ongoing epidemiological and entomological studies are aimed at further characterizing malaria hotspots in the area in order to provide insights into the residual transmission dynamics of the northwestern Peruvian coast.
